# MARCH5 promotes aerobic glycolysis to facilitate ovarian cancer progression via ubiquitinating MPC1

**DOI:** 10.1007/s10495-024-01962-5

**Published:** 2024-04-13

**Authors:** Ying Xu, Shuhua Zhao, Yujie Shen, Yuanfeng Li, Yinghui Dang, Fenfen Guo, Zhihao Chen, Jia Li, Hong Yang

**Affiliations:** Department of Gynaecology and Obstetrics, Xijing Hospital, Air Force Medical University, Xi’an, China

**Keywords:** Membrane associated ring finger protein 5, Growth, Metastasis, Glycolysis, OC

## Abstract

MARCH5 is a ring-finger E3 ubiquitin ligase located in the outer membrane of mitochondria. A previous study has reported that MARCH5 was up-regulated and contributed to the migration and invasion of OC cells by serving as a competing endogenous RNA. However, as a mitochondrial localized E3 ubiquitin ligase, the function of MARCH5 in mitochondrial-associated metabolism reprogramming in human cancers remains largely unexplored, including OC. We first assessed the glycolysis effect of MARCH5 in OC both in vitro and in vivo. Then we analyzed the effect of MARCH5 knockdown or overexpression on respiratory activity by evaluating oxygen consumption rate, activities of OXPHOS complexes and production of ATP in OC cells with MARCH5. Co-immunoprecipitation, western-blot, and in vitro and vivo experiments were performed to investigate the molecular mechanisms underlying MARCH5-enhanced aerobic glycolysis s in OC. In this study, we demonstrate that the abnormal upregulation of MARCH5 is accompanied by significantly increased aerobic glycolysis in OC. Mechanistically, MARCH5 promotes aerobic glycolysis via ubiquitinating and degrading mitochondrial pyruvate carrier 1 (MPC1), which mediates the transport of cytosolic pyruvate into mitochondria by localizing on mitochondria outer membrane. In line with this, MPC1 expression is significantly decreased and its downregulation is closely correlated with unfavorable survival. Furthermore, in vitro and in vivo assays revealed that MARCH5 upregulation-enhanced aerobic glycolysis played a critical role in the proliferation and metastasis of OC cells. Taken together, we identify a MARCH5-regulated aerobic glycolysis mechanism by degradation of MPC1, and provide a rationale for therapeutic targeting of aerobic glycolysis via MARCH5-MPC1 axis inhibition.

**Supplementary Information:**

The online version contains supplementary material available at 10.1007/s10495-024-01962-5.

## Background

Metabolic reprogramming, characterized by increased aerobic glycolysis (also called Warburg effect) even in the presence of oxygen, has been known as a hallmark of malignancy, which is critical for cancer cells to support their energy demand and biomass synthesis required for hyperproliferation, invasion and metastasis [1, 2]. However, the understanding of mechanism mediating increased aerobic glycolysis in cancer remains incomplete.

Mitochondrial pyruvate carrier (MPC) is a heterodimer composed of MPC1 and MPC2, which functions as the junction between cytoplasmic glycolysis and mitochondrial oxidative phosphorylation by regulating pyruvate transport from cytoplasm to mitochondrion [3]. During recent years, studies have reported that the expression of MPC1 was decreased in several types of human cancers, which result in suppression of MPC activity and cancer progression [4–8]. However, the mechanism underlying the downregulation of MPC1 remains largely unknown. Additionally, the role of MPC in OC has not yet been studied.

Membrane associated RING finger protein 5 (MARCH5) is a ring-finger E3 ubiquitin ligase located in the outer membrane of mitochondria [9]. Previous studies have reported the regulation of mitochondrial dynamics by MARCH5 via ubiquitinating Mfn1, Drp1 and Fis1 [10]. In addition, MARCH5 participates in mitochondrial quality control by recruiting Parkin to impaired mitochondria for nonselective ubiquitylation [11]. A previous study in ovarian cancer has revealed a tumor-promoting function of MARCH5 in cell migration and invasion by serving as a competing endogenous RNA [12]. However, as a mitochondrial localized E3 ubiquitin ligase, the function of MARCH5 in mitochondria-associated metabolism reprogramming in human cancers remains largely unexplored, including OC.

Here, we report that the abnormal upregulation of MARCH5 promotes aerobic glycolysis by ubiquitinating and degrading MPC1 in OC cells. Our findings identify a MARCH5-regulated aerobic glycolysis mechanism in OC tumorigenesis and provide a rationale for therapeutic targeting of aerobic glycolysis via MARCH5 inhibition.

## Materials and methods

### Clinical tissue samples

A total of 205 pairs of tumor and adjacent non-tumor tissue samples were obtained from OC patients at the first affiliated hospital of Air Force Medical University (Xi’an, China) as previously described [13]. All tissue samples were collected with the informed consent from the patients, who are diagnosed with OC by postoperative pathology. The use of human OC tissues was approved by the Ethics Committee at the first affiliated hospital of Air Force Medical University.

### Cell culture and transfection assays

Human OC cell lines A2780, OVCAR3, CAOV3, TOV112D and normal ovarian epithelial cell line ISO80 were maintained in DMEM culture medium supplemented with 10% FBS (GIBCO, USA) at 37 °C in a humidified incubator with 5% CO_2_.

For overexpression of MARCH5 or MPC1, their cDNAs was generated by PCR and then cloned into pcDNA™3.1(C) vector (Invitrogen, V790–20). For knockdown of MARCH5 or MPC1, corresponding siRNAs were purchased from RiboBio (Guangzhou, China). Transfection was conducted using Lipofectamine 2000 following the manufacturer’s protocol.

### Short- and long-term cell proliferation assays

The CCK-8 cell viability and colony formation assays were used for determination of short-term or long-term cell proliferation, respectively. For CCK-8 cell viability assay, 1 × 10^3^ OC cells were seeded into 96-well plates. After incubation with CCK-8 solution (10 µL) for 2 h, the absorbance at 490 nm was measured at 37 °C with a Bio-Rad’s microplate reader. For colony formation assay, 1 × 10^3^ OC cells were plated into 6-well plates and cultured for 14 days. Colonies were fixed, stained with crystal violet and counted.

### RNA extraction and quantitative real-time PCR

An RNA extraction from cell lines or tissue samples was performed by using trizol reagent. Reverse transcription of RNA into cDNA was conducted using the Revert Aid First Strand cDNA Synthesis Kit. Quantitative real-time PCR analysis was performed with SYBR Green I. Results were normalized to β-actin levels as endogenous control. The primer sequences were provided in the Table S1.

### Immunohistochemistry (IHC) staining

Tissues were fixed in formalin for 24 h and embedded with paraffin. IHC staining was conducted following the instructions of the IHC Kit (Maixin-Bio Co., Fuzhou, China) as previously described [13]. Primary antibodies used in this study were provided in Table S2. Staining images were acquired using a microscope (Olympus).

### In vivo nude mice experiments

Four-to five-week-old female athymic nude mice were used in this study. The mice were housed in specific pathogen-free (SPF) environment on a 12-h light/dark cycle at temperature 18–24 °C. All animal procedures were performed in accordance with the guidelines of the Institutional Committee for the Ethics of Animal Care and Treatment in the Air Force Medical University. For xenograft tumor growth model, 5 × 10^6^ OC cells were injected into the flanks of the mice. A caliper was used for the measurement of tumor volumes every week for five weeks. For in vivo metastatic assay, 4 × 10^6^ OC cells were intravenously injected into the tail vein of the nude mice. The lungs were collected for HE staining five weeks post cells injection.

### Cell cycle and apoptosis analyses

A cell cycle detection kit and an annexin V (FITC-conjugated) apoptosis kit purchased from US Everbright inc were used for determinations of cell cycle or apoptosis analyses, respectively, following their manufacturer’s protocols as previously described [13]. The results were analyzed by a flow cytometry (Beckman, Fullerton, CA).

### Metabolomics analysis

For metabolites extraction, OC cells were collected in PBS buffer and frozen in liquid nitrogen. Cells were then mixed with 400 µL of 80% methanol aqueous solution, and centrifugated at 13,000 rpm at 4 °C for 20 min. The supernatant was collected and lyophilized for metabolomics analysis using LC-MS.

### Extracellular acidification rate (ECAR) and oxygen consumption rate (OCR) assays

The ECAR and OCR were measured in a XF96 Extracellular Flux Analyzer (Seahorse Bioscience). Briefly, a total of 1.0 × 10^4^ OC cells were plated in an XF96 plate and incubated overnight. One hour before the assay, the cell culture medium was replaced by analysis media. ECAR was determined following the manufacturer’s instructions. An XF Glycolysis Stress Test Kit or a Cell Mito Stress Test Kit was used to measure the glycolytic capacity or cellular mitochondrial function following their manufacturer’s instructions.

### Measurement of glucose uptake, lactate production and ATP levels

Glucose uptake and lactate production were measured using a glucose uptake colorimetric assay kit or a lactate colorimetric assay kit purchased from Nanjing jiancheng Bioengineering institute (Nanjing, China) according to their manufacturer’s protocol as previously described [13]. ATP level was measured with an ATP Determination Kit (Thermo Fisher Scientific) according to the manufacturer’s protocol.

### Statistics

SPSS 17.0 software (SPSS, Chicago, IL, USA) was used for statistical analyses. Student’s t-test was used for comparison between two groups, and ANOVA was used for comparison among three or more groups. Kaplan-Meier method and log-rank test were used for survival analysis. P value less than 0.05 was considered statistically significant.

## Results

### MARCH5 potentiates glycolytic metabolism in OC

**Fig. 1:**
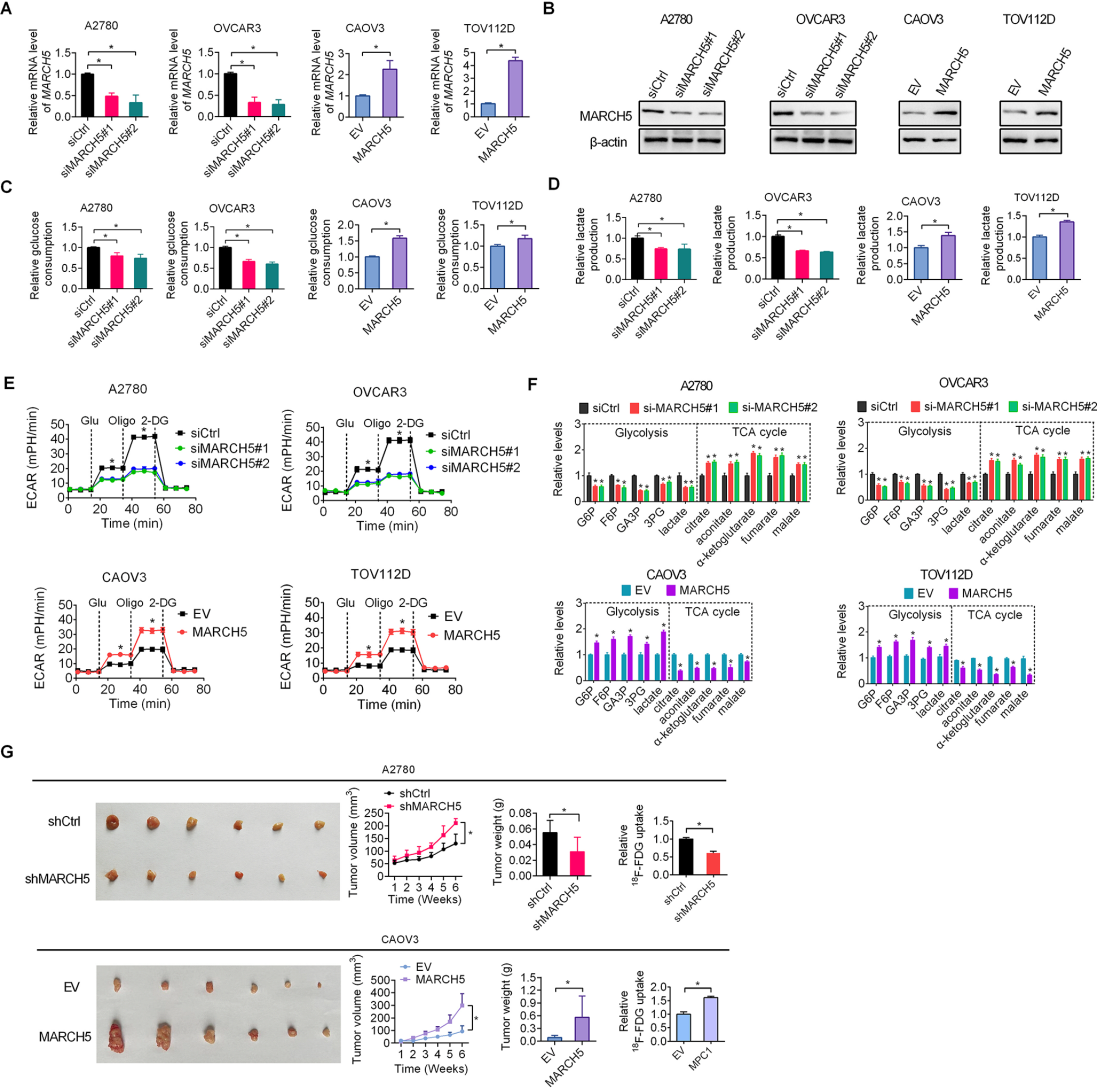
MARCH5 potentiates glycolytic metabolism in OC. **(A and B)** MARCH5 expression was detected by qRT-PCR and western blotting assays in indicated OC cells with MARCH5 knockdown or overexpression. **(C and D)** Glucose uptake and lactate generation were evaluated in indicated OC cells with MARCH5 knockdown or overexpression. **(E)** Extracellular acidification rate (ECAR) was measured in indicated OC cells with MARCH5 knockdown or overexpression. **(F)** The intracellular levels of glycolytic and TCA-circulating metabolites were measured in OC cells with MARCH5 knockdown or overexpression. **(G)** Measurement of 18 F-FDG uptake in indicated subcutaneous tumors developed from OC cells with MARCH5 knockdown or overexpression

To explore whether MARCH5 regulates glucose metabolism in OC, we knocked-down MARCH5 expression in A2780 and OVCAR3 with relative higher MARCH5 expression level and overexpressed MARCH5 in CAOV3 and TOV112D cells with relative low MARCH5 expression level (Fig. 1A and B, supplementary Fig S1).

Knockdown of MARCH5 resulted in a significant decrease of glucose uptake and lactate generation in A2780 and OVCAR3 cells, whereas overexpression of MARCH5 enhanced glucose uptake and lactate generation in CAOV3 and TOV112D cells (Fig. 1C and D). To better determine the role of MARCH5 in the promotion of glycolysis in OC, glucose-induced extracellular acidification rate (ECAR), a proxy for the rate of glycolysis and glycolytic capacity, was further measured. The results showed that knockdown of MARCH5 resulted in a significant decrease in ECAR, while overexpression of MARCH5 led to a significant increase in ECAR (Fig. 1E). To confirm the regulation of glycolysis by MARCH5, the levels of intracellular metabolites were quantified. MARCH5 knockdown decreased abundance of glycolytic metabolites such as glucose 6-phosphate (G6P), fructose 6-phosphate (F6P), glyceraldehyde 3-phosphate (GA3P), 3-phosphoglycerate (3PG) and lactate, while increased abundance of TCA-circulating metabolites such as citrate, aconitate, α-ketoglutarate, fumarate, malate. Conversely, MARCH5 overexpression increased abundance of glycolytic intermediates but reduced TCA-circulating metabolites (Fig. 1F). To further corroborate these results, ^18^F-deoxyglucose (^18^F-FDG) uptake assay was employed in subcutaneous tumors. The results showed that MARCH5 knockdown markedly suppressed tumor growth and glucose uptake, whereas opposite results were obtained upon MARCH5 overexpression (Fig. 1G). Taken together, these results indicate that MARCH5 contributes to glycolysis in OC both in vitro and in vivo.

### MARCH5 suppresses mitochondrial OXPHOS activity in a morphology change independent manner

**Fig. 2:**
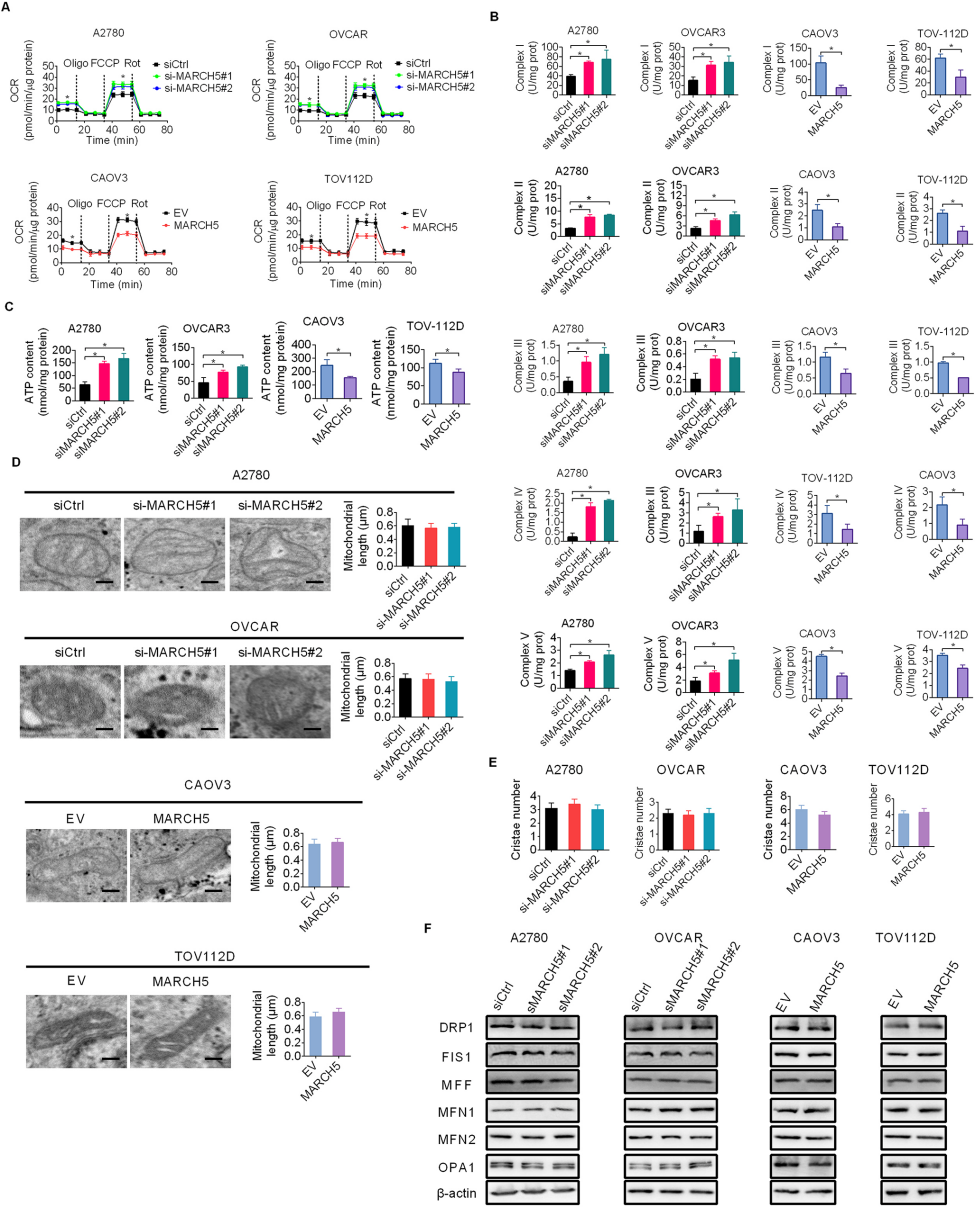
MARCH5 suppresses mitochondrial OXPHOS activity in a morphology change independent manner. **(A-C)** Oxygen consumption rate OCR (**A**), activities of OXPHOS complexes (**B**) and production of ATP (**C**) were evaluated in OC cells with MARCH5 knockdown or overexpression. (**D** and **E**) Electron microscopy analysis for mitochondrial length (**D**) and cristae number (**E**) were conducted in OC cells with MARCH5 knockdown or overexpression. Scale bar, 0.1 μm. (**F**) Western blotting assay for expressions of mitochondrial fission and fusion regulators in OC cells with MARCH5 knockdown or overexpression

Since increased aerobic glycolysis always accompanied by impaired mitochondrial respiration [14, 15], we therefore analyzed the effect of MARCH5 knockdown or overexpression on respiratory activity by evaluating oxygen consumption rate (OCR), activities of OXPHOS complexes and production of ATP in OC cells with MARCH5. Obvious increases in OCR (Fig. 2A), activities of OXPHOS complexes (Fig. 2B) and production of ATP (Fig. 2C) were observed in MARCH-knockdown A2780 and OVCAR3 cells, while opposite mitochondrial respiratory phenotypes were obtained in MARCH5-overexpression CAOV3 and TOV112D cells (Fig. 2A and C), indicating that MARCH5 suppresses OXPHOS activity in OC cells. Given that MARCH5 has been reported to participate in mitochondrial morphology remodeling by ubiquitinating mitochondrial fission and fusion factors, we explored whether MARCH5 suppress mitochondrial OXPHOS by influencing mitochondrial morphology. Unexpectedly, no obvious alterations in mitochondria length and cristae number were observed when MARCH5 was either knocked-down or over-expressed in OC cells (Fig. 2D and E), which is contrary to our original thought that MARCH5 would influence mitochondrial morphology in OC cells. In line with this, no changes in the expressions of mitochondrial fission and fusion factors were found when MARCH5 was knocked-down or overexpressed (Fig. 2F). Together, these results suggest that MARCH5 suppresses mitochondrial OXPHOS activity in a morphological change independent manner.

### MARCH5 interacts with MPC1 to promote its ubiquitination and degradation

**Fig. 3:**
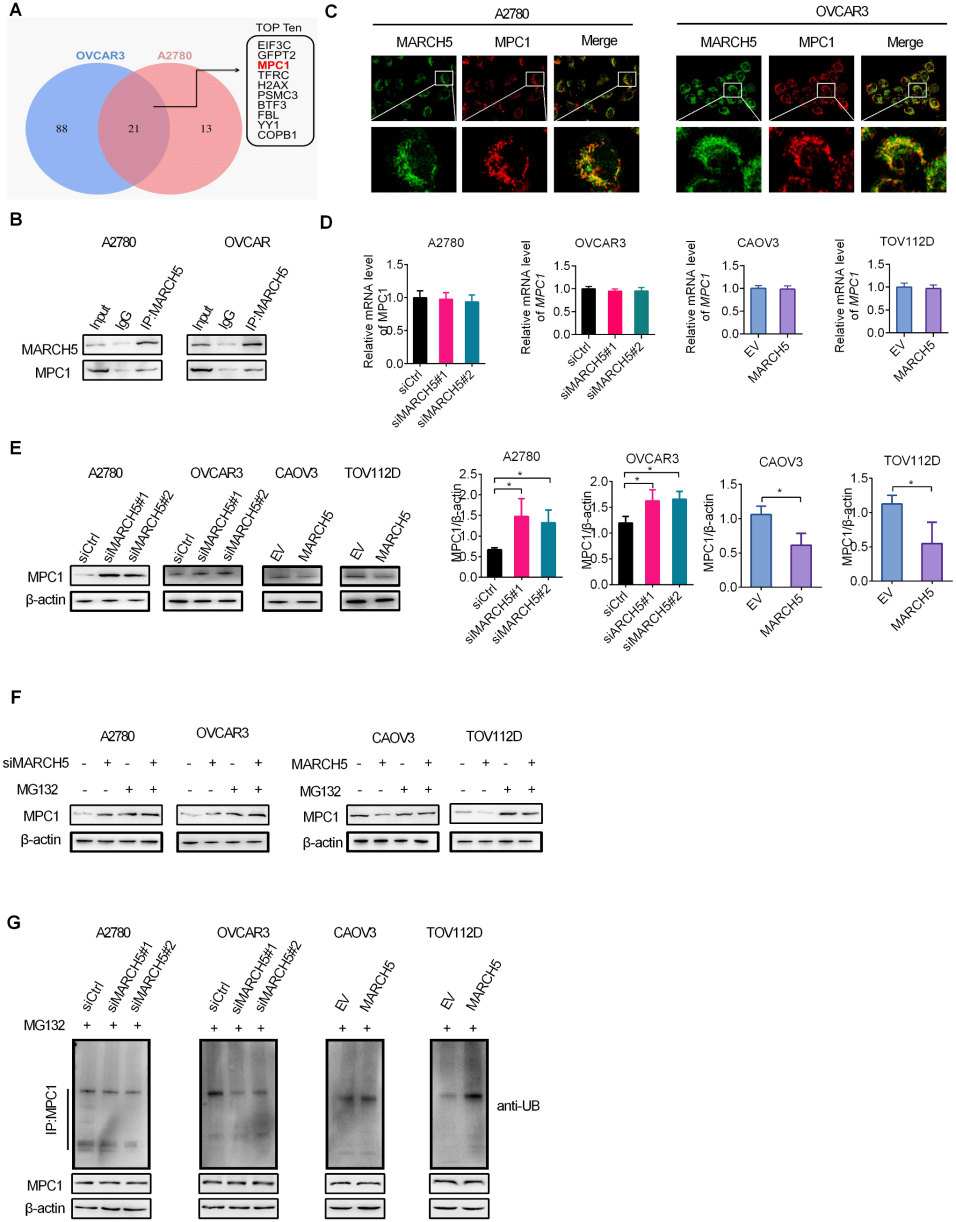
MARCH5 interacts with MPC1 to promote its ubiquitination and degradation. **(A)** IP-MS analysis for MARCH5-interacting proteins in OC cells. **(B)** Co-IP assay for interaction between MARCH5 and MPC1 in OC cells. **(C)** Double immunofluorescent staining of MARCH5 and MPC1 in OC cells. (**D** and **E**) The mRNA and protein expression levels of MPC1 were detected by qRT-PCR and western blotting assays in OC cells with MARCH5 knockdown or overexpression. **(F)** The protein levels of MARCH5 and MPC1 were detected by western blotting assay in indicated OC cells in the presence of protein degradation inhibitor MG132. **(G)** The protein expression and ubiquitination levels of MPC1 were detected by western blotting assay in indicated OC cells in the presence of proteasome inhibitor MG132

To gain insight into the molecular mechanistic basis by which MARCH5 shifts energy production from mitochondrial respiration towards aerobic glycolysis in OC, co-immunoprecipitation (Co-IP) and mass spectrometry assays were conducted to identify potential aerobic glycolysis-associated substrates of MARCH5 in OC cells. Notably, mitochondrial pyruvate carrier 1 (MPC1), a specific mitochondrial inner membrane-localized carrier achieving a crucial role in linking glycolysis to oxidative phosphorylation, was identified as a potential interacting partner with MARCH5 (Fig. 3A). Co-IP assays showed that MARCH5 directly interacted with MPC1 in OC cells (Fig. 3B). In agreement with this, double immunofluorescent staining assay also showed a clear colocalization of MARCH5 and MPC1 in OC cells (Fig. 3C). Additionally, we found that the protein expression level of MPC1, but not mRNA expression level, was elevated in A2780 and OVCAR3 cells after knockdown of MARCH5, while opposite results were observed in CAOV3 and TOV112D cells upon MARCH5 overexpression (Fig. 3D and E). The above results suggest that MARCH5 may downregulate MPC1 by promoting its ubiquitination and degradation. To test whether MARCH5 promotes the ubiquitination of MPC1, OC cells were treated with MG132 to inhibit protein degradation. We found that the effect of MARCH5 on MPC1 expression was markedly abolished when OC cells were treated with proteasome inhibitor MG132 (Fig. 3F). In agreement with this, the ubiquitination of MARCH5 was significantly decreased by MARCH5 knockdown and increased by MARCH5 overexpression (Fig. 3G). Together, these data indicate that MARCH5 interacts with MPC1 to promote its ubiquitination and degradation in OC cells.

### MPC1 is downregulated and correlates with poorer patient survival in OC

**Fig. 4:**
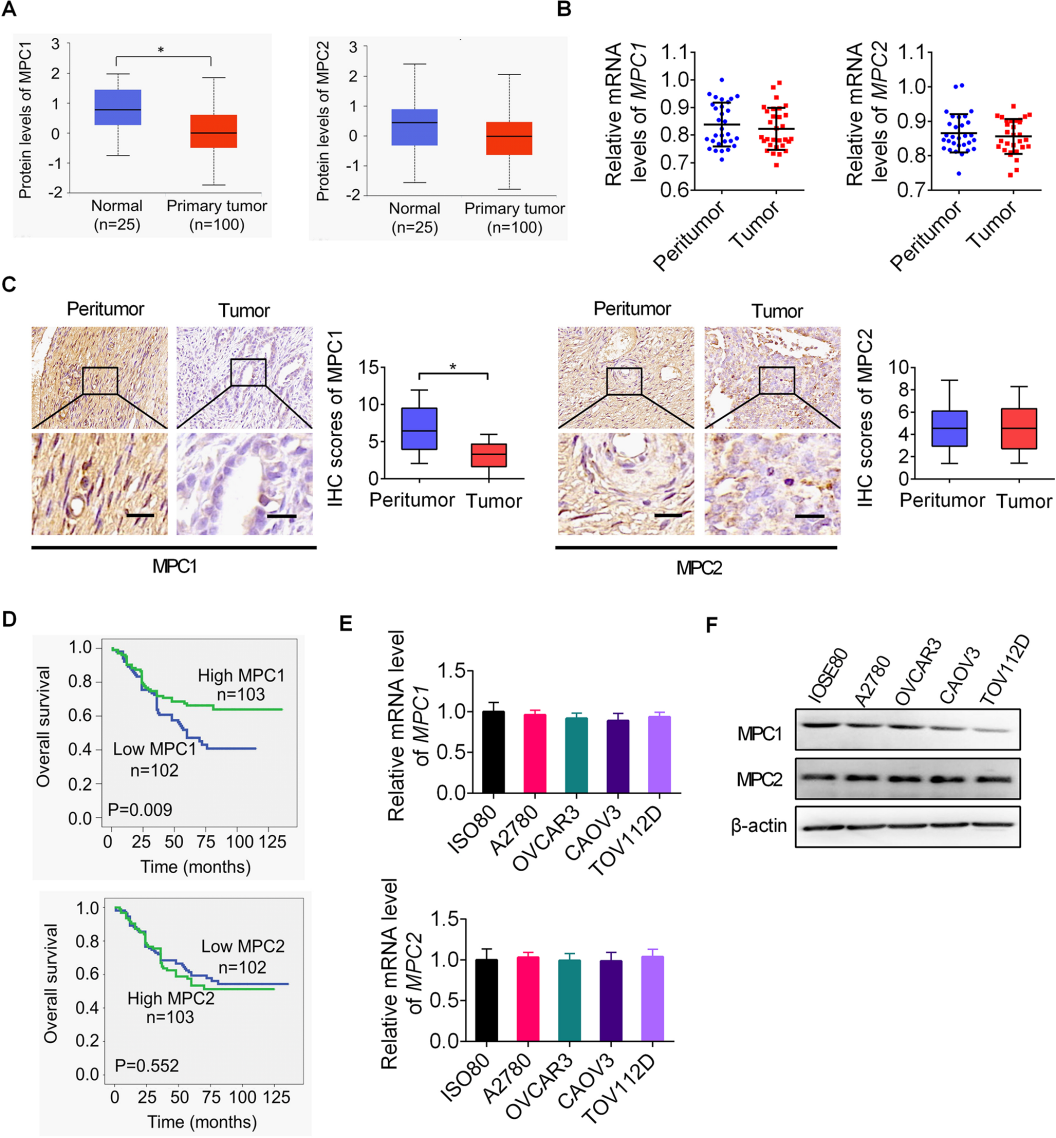
MPC1 is downregulated and correlates with poorer patient survival in OC. **(A)** The protein expression levels of MPC-1 and MPC-2 were analyzed using the online UALCAN database. **(B)** The expressions of MPC1 and MPC2 at mRNA level were determined by qRT-PCR analysis in 30 paired OC and adjacent non-tumor tissues. **(C)** The expressions of MPC1 expression at protein level was determined by IHC staining assay in another 205 paired OC and adjacent non-tumor tissues. **(D)** Kaplan–Meier survival analysis for OC patients with different protein expression levels of MPC1 or MPC2. **(E and F)** qRT-PCR (**E**) and western blotting (**F**) analyses were undertaken for expressions of MPC1 and MPC2 in five OC cell lines and one normal ovarian epithelial cell line

Downregulation of MPC1 or MPC2 has been observed in several types of human cancers such as kidney, cholangiocarcinoma and breast cancer [4, 16–18]. However, the expressions and clinical implications of MPC1 and MPC2 in OC have not been studied previously. Using the online UALCAN, significantly decreased MPC1 expression level was observed in OC tissues as compared with normal tissues, while the expression level of MPC-2 was comparable in OC and normal tissues (Fig. 4A). The expressions of MPC1 and MPC2 were then determined in 30 paired tumor and adjacent non-tumor tissues of OC using qRT-PCR analysis, as well as in another 205 paired tumor and adjacent non-tumor tissues of OC using IHC analysis. MPC1 expression shows no significant difference between tumor and adjacent non-tumor tissues of OC at mRNA level (Fig. 4B), while its protein expression level was significantly decreased in OC as compared with adjacent non-tumor tissues (Fig. 4C). However, MPC-2 expression level was comparable at both mRNA and protein level in tumor and adjacent non-tumor tissues of OC (Fig. 4B and C). Survival analysis based on IHC staining results indicated that OC patients with low MPC-1 expression had worse survival than those with high MPC-1 expression, while no association was found between the expression of MPC-2 and OC patients’ survival (Fig. 4D). Moreover, the expression level of MPC-1 was also negatively associated with lager tumor size and lymph nodes metastasis (Table S3). To provide more support, the expressions of MPC1 and MPC2 were also determined in OC and normal ovarian epithelial cell lines. In keeping with the findings in OC tissues, the mRNA expression level of MPC1 does not significantly change between ovarian cancer cell lines and normal ovarian epithelial cell line, while the protein expression level of MPC1 was also markedly lower in OC cell lines than in normal ovarian epithelial cell line (Fig. 4E and F). Collectively, these results demonstrate that the protein expression level of MPC1 is downregulated in OC, which correlates with poorer patient survival.

### Downregulation of MPC1 promotes growth and metastasis of OC

**Fig. 5:**
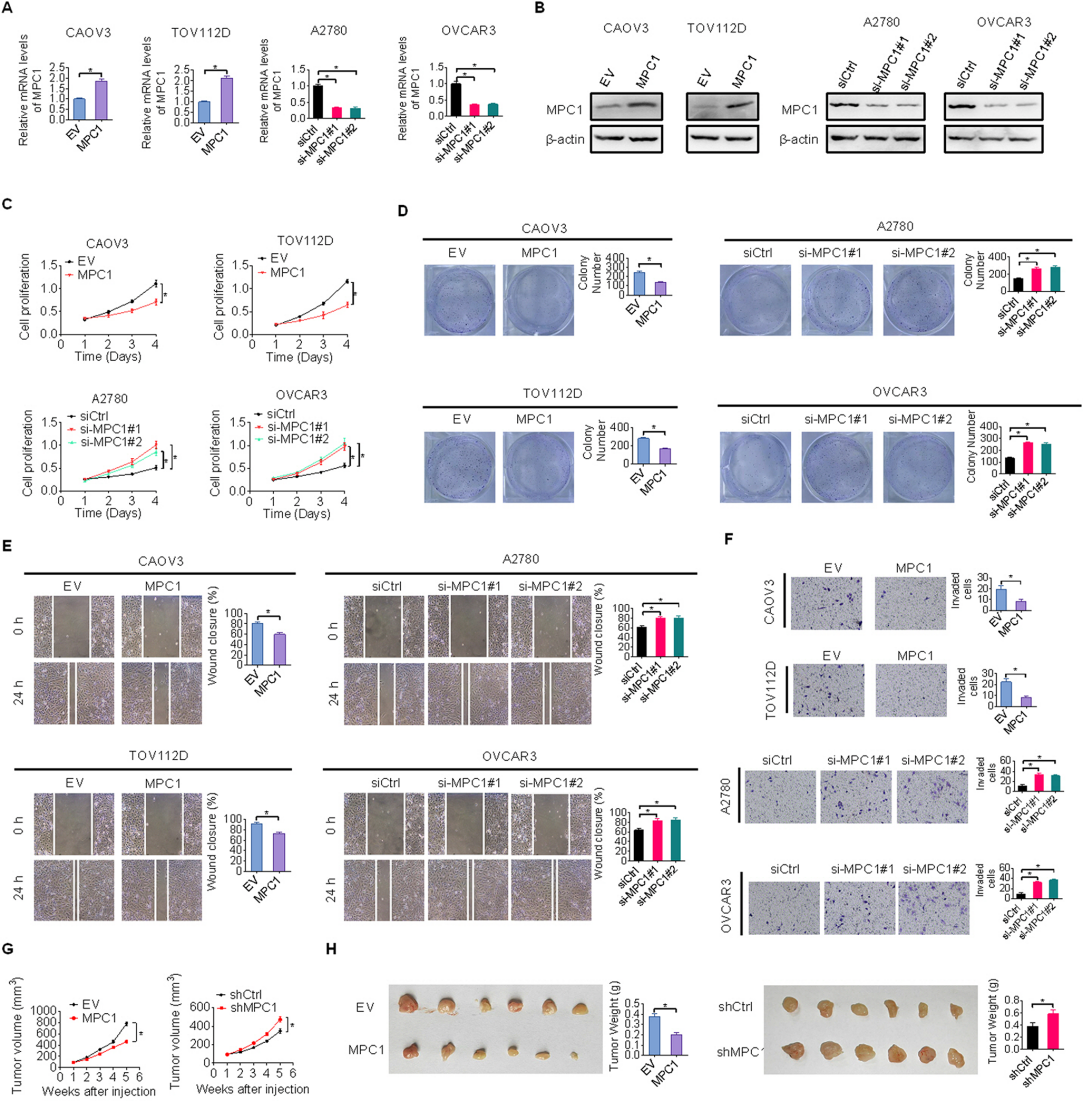
Downregulation of MPC1 promotes growth and metastasis of O**C.** (**A** and **B**) qRT-PCR (**A**) and western blot (**B**) analyses of MPC1 expression in OC cells with MPC1 knockdown or overexpression. (**C** and **D**) Cell proliferation and colony-forming abilities were analyzed using CCK-8 and colony formation assays in OC cells with indicated treatments. (**E** and **F**) Cell migration and invasion abilities were analyzed using wound healing and transwell invasion assays in OC cells with indicated treatments. (**G** and **H**) Tumor images and growth curves (**G**), and their weight (**H**) were compared in nude mice injected with MPC1-overexpressing CAOV3 cells or MPC1-depleted A2780 cells

Since MPC1 is downregulated in OC, and this downregulation correlates with poor patient survival, we next sought to determine the functional roles of MPC1 in OC progression. By performing gain-of-function analysis in CAOV3 and TOV112D cells and loss-of-function in A2780 and OVCAR3 cells (Fig. 5A and B), we found that forced expression of MPC1 markedly suppressed proliferation and colony formation abilities in CAOV3 and TOV112D cells. Conversely, knockdown of MPC1 by two independent MPC1 siRNAs promoted proliferation and colony-forming abilities in A2780 and OVCAR3 cells (Fig. 5C and D). We also explored the function of MPC1 in metastasis of OC using wound healing and transwell invasion assays. The migration and invasion abilities were clearly suppressed in CAOV3 and TOV112D cells when MPC1 was overexpressed, whereas opposite results were obtained when MPC1 was knocked-down in A2780 and OVCAR3 cells (Fig. 5E and F). To further explore the function of MPC1 in OC cells in vivo, MPC1-overexpressing CAOV3 and MPC1-depleted A2780 cells were subcutaneously implanted into four- to five-week-old female Balb/c nude mice. The mice injected with MPC1-overexpressing CAOV3 cells had clearly smaller tumor volumes and weights than those injected with control CAOV3 cells. By contrast, knockdown of MPC1 enhanced the tumorigenic ability of A2780 cells (Fig. 5G and H). Together, the above data confirm that MPC1 downregulation plays an important role in the promotion of tumor growth and metastasis.

### MARCH5 promotes aerobic glycolysis by downregulating MPC1 in OC cells

**Fig. 6:**
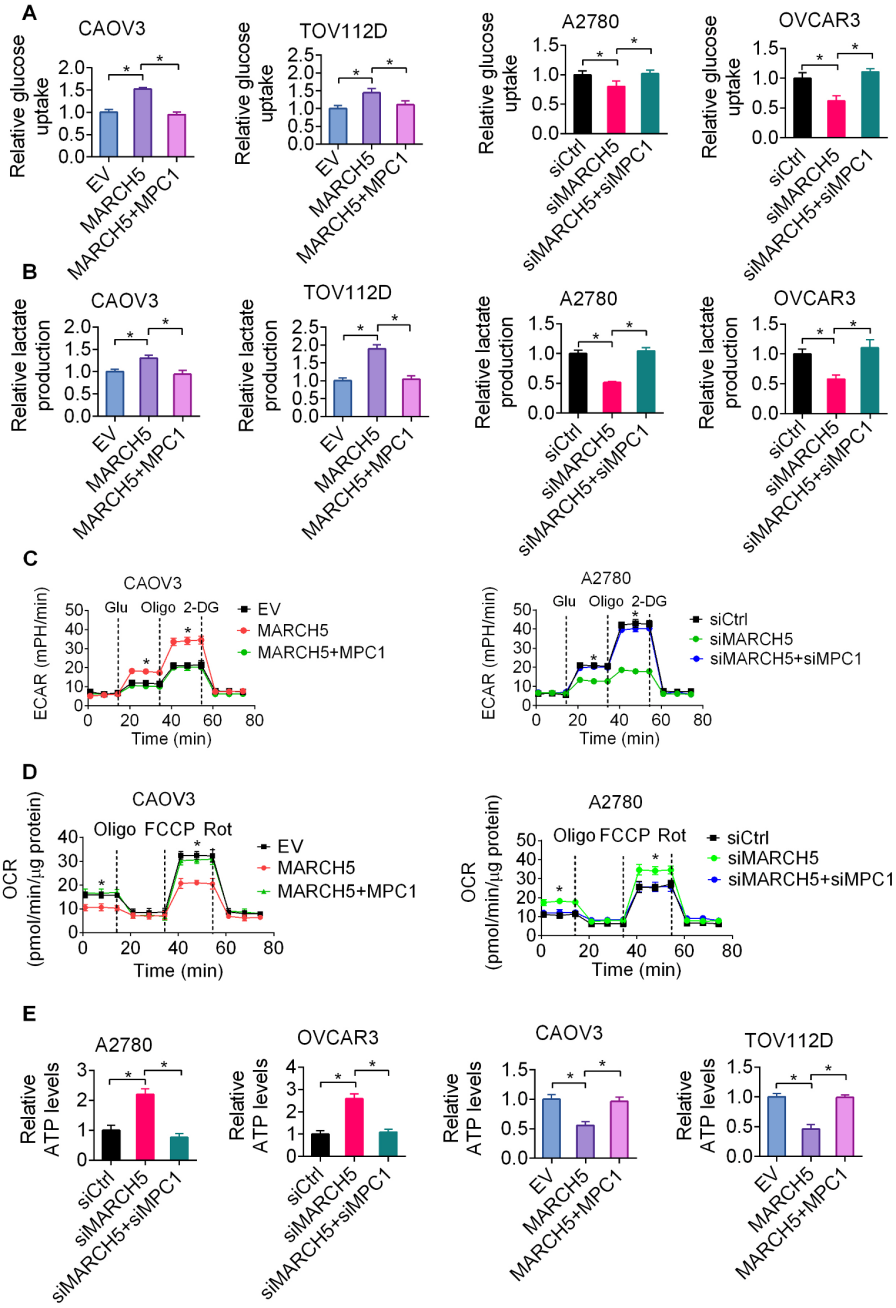
MARCH5 promotes aerobic glycolysis by downregulating MPC1 in OC cells. **(A and B)** Glucose uptake (**A**) and lactate generation (**B**) were evaluated in OC cells with indicated treatment. **(C)** Extracellular acidification rate (ECAR) was measured in OC cells with indicated treatment. (**D**) Oxygen consumption rate (OCR) was measured in OC cells with indicated treatment. **(E)** ATP production was measured in OC cells with indicated treatment

Given that MPC functions as the junction between cytoplasmic glycolysis and mitochondrial oxidative phosphorylation by regulating pyruvate transport from cytoplasm to mitochondrion, we therefore determined whether MARCH5 promotes aerobic glycolysis in OC cells by downregulating MPC1 expression. The results of a series of rescue experiments showed that increased aerobic glycolysis by MARCH5 overexpression was obviously attenuated by forced expression of MPC1 in CAOV3 and TOV112D cells, whereas suppressed aerobic glycolysis by MARCH5 knockdown was clearly rescued by downregulation of MPC1 in A2780 and OVCAR3 cells, as evaluated by glucose uptake (Fig. 5A), lactate generation (Fig. 6B), ECAR (Fig. 6C), OCR (Fig. 6D) and ATP production (Fig. 6E) assays, indicating that MARCH5 promotes aerobic glycolysis by downregulating MPC1 in OC cells.

### MPC1 downregulation-enhanced aerobic glycolysis contributed to the oncogenic functions of MARCH5 in OC

**Fig. 7:**
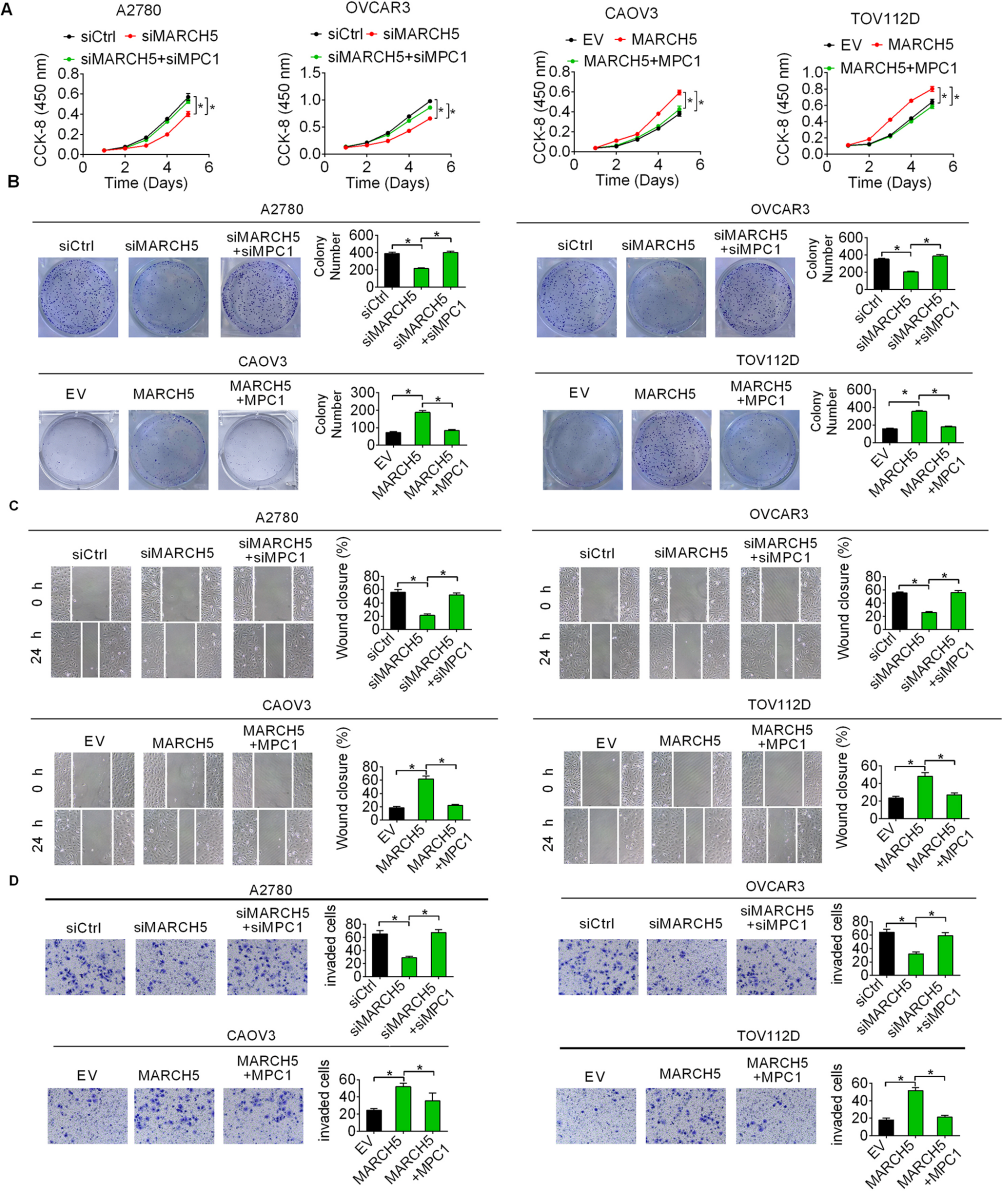
MPC1 downregulation-enhanced aerobic glycolysis contributed to the oncogenic functions of MARCH5 in OC. **(A and B)** The proliferation and colony formation abilities were analyzed in OC cells with indicated treatment. **(C and D)** The migration and invasion abilities were analyzed in OC cells with indicated treatment

Given the crucial roles played by aerobic glycolysis in tumorigenesis, we explored whether MARCH5 promote the growth and metastasis of OC cells by activating aerobic glycolysis. We found that activation of aerobic glycolysis by MPC1 silencing markedly restored the proliferation (Fig. 7A), colony formation (Fig. 7B), migration (Fig. 7C) and invasion (Fig. 7D) of OC cells suppressed by MARCH5 knockdown. By contrast, suppression of aerobic glycolysis via MPC-1 overexpression markedly impaired the proliferation, colony formation, migration and invasion of OC cells promoted by MARCH5 overexpression (Fig. 7A and D). These results suggest that MPC1 downregulation-enhanced aerobic glycolysis is essential for the oncogenic functions of MARCH5 in OC.

## Discussion

Metabolic reprogramming, particularly increased aerobic glycolysis, has been well recognized as a hallmark of cancer, which is requirement for tumor growth and metastasis. MARCH5 is an E3 ubiquitin ligase located in the outer membrane of mitochondria, which has been reported to be up-regulated and play a crucial role in the migration and invasion of OC cells by serving as a competing endogenous RNA [12]. However, as a mitochondrial-localized E3 ubiquitin ligase, the role of MARCH5 in mitochondrial metabolism reprogramming in human cancers remains largely unknown, including OC. In our study, we demonstrate that MARCH5 functions as a key regulator of aerobic glycolysis by ubiquitinating MPC1 to promote ovarian cancer progression.

MPC plays a necessary role in transporting pyruvate from cytoplasm into mitochondrion, which links glycolysis to mitochondrial respiration [19]. It has been reported that the expression of MPC1 and MPC2 is frequently decreased in several types of cancer. For example, it was reported in colon cancer that both expressions of MPC1 and MPC2 are decreased in tumor tissues as compared with normal counterparts [20]. Although MPC is formed by two subunits, previous studies indicated that the expression MPC1 subunit is specifically dysregulated and associated with poor prognosis in several cancers, including cholangiocarcinoma and prostate cancer [6, 21]. However, the expression and functions of MPC-1 in OC remains unexplored. Here, we demonstrate that the expression of MPC1 but not MPC2 is significantly decreased, which correlates with poor patient survival.in OC. In addition, downregulation of MPC1 promoted the proliferation and metastasis of OC cells. Consistent with our findings in OC, MPC1 downregulation-mediated suppression of MPC also promoted colon cancer cell growth both in vitro and in vivo [22]. On the contrary, in other types of cancer, upregulation of MPC was reported to exert an oncogenic function [17, 18]. For example, it was reported that MPC1 upregulation promoted the metastasis of cholangiocarcinoma by reversing Warburg effect. These findings suggest that the function of MPC may vary considerably among different malignancies.

Previous studies have revealed that MPC downregulation mainly occurs at the transcriptional level. In prostate cancer, MPC1 expression was repressed by the transcriptional repressor of chicken ovalbumin upstream promoter-transcription factor II (COUP-TFII) [6]. In pancreatic cancer, MPC1 was also reported to be transcriptionally suppressed by Lysine demethylase 5 A (KDM5A)-mediated demethylation H3K4 [23]. Besides, downregulation of peroxisome proliferator-activated receptor-gamma co-activator (PGC)-1α was also reported to contribute to MPC1 silencing at transcriptional level in breast cancer [24], renal cell carcinoma [16] and cholangiocarcinoma [17]. In addition to the regulation of MPC expression at transcriptional level, MPC can also be regulated at post-transcriptional level. In colon cancer cells, deacetylation of MPC1 at lysine 45 and 46 by sirt3 enhances its activity [25]. Similarly, MPC2 acetylation at lysine 19 and 26 significantly decreased the activity of MPC in diabetic heart [26]. Our data revealed MARCH5, a critical mitochondrial-localized E3 ubiquitin ligase, as a new critical regulator of MPC by directly interacting with MPC1 to promote its ubiquitination and degradation in OC cells.

MARCH5, also known as mitochondrial ubiquitin ligase (MITOL), is a transmembrane protein localized in the outer membrane of mitochondrial. Studies have demonstrated that MARCH5 was significantly up-regulated and contributed to the malignant progression in several types of human cancers, including breast cancer [27], nasopharyngeal carcinoma [28]and liver cancer [29]. In OC, MARCH5 was also up-regulated and its RNA contributes to the migration and invasion of OC cells by serving as a competing endogenous RNA [12]. However, previous studies mainly focused on the role of MARCH5 in morphology remodeling and quality control of mitochondria [11, 30]. As a critical E3 ubiquitin ligase located in mitochondrial, little is known about the function of MARCH5 in mitochondrial metabolism reprogramming. Our data demonstrate that MARCH5 promotes aerobic glycolysis and inhibits mitochondrial OXPHO in OC via ubiquitinating and degrading MPC1. Furthermore, we found that suppression of MPC1-regulated aerobic glycolysis was critical for the oncogenic functions of MARCH5 in the promotion of proliferation and metastasis of OC cells.

In conclusion, our present study demonstrates mitochondria outer membrane-localized E3 ubiquitin ligase MARCH5 as a novel regulator of aerobic glycolysis by ubiquitinating and degrading MPC1 in OC. Therefore, MARCH5/MPC1 signaling may serve as a potential therapeutic target to normalize deranged glucose metabolism to suppress ovarian cancer progression.

### Electronic supplementary material

Below is the link to the electronic supplementary material.

Supplementary Material 1

## Data Availability

The data generated during the current study are available from the corresponding author upon reasonable request.

## Abbreviations

MPC: Mitochondrial pyruvate carrier
MARCH5: Membrane associated RING finger protein 5
SPF: specific pathogen-free
ECAR: Extracellular acidification rate
OCR: oxygen consumption rate
G6P: glucose 6-phosphate
F6P: fructose 6-phosphate
GA3P: glyceraldehyde 3-phosphate
3PG: 3-phosphoglycerate
CHX: cycloheximide
COUP-TFII: chicken ovalbumin upstream promoter-transcription factor II
KDM5A: Lysine demethylase 5 A
PGC: proliferator-activated receptor-gamma co-activator
MITOL: mitochondrial ubiquitin ligase
