# Supplementary material for: MARCH5 promotes aerobic glycolysis to facilitate ovarian cancer progression via ubiquitinating MPC1
Source: Apoptosis. 2024 Apr 13;29(7-8):1232–45. doi: 10.1007/s10495-024-01962-5 (PMC11263418; doi:10.1007/s10495-024-01962-5)
Supplement: Supplementary file 1 — Supplementary Material 1 [file 10495_2024_1962_MOESM1_ESM.docx]

**Supplemental information**

**MARCH5 promotes aerobic glycolysis to facilitate ovarian cancer progression via ubiquitinating MPC1**

**Supplemental tables**

**Table S1.** Sequence of primers used for qRT-PCR analysis

| *MARCH5* | forward primer | GTCCAGTGGTTTACGTCTTGG |
| --- | --- | --- |
|  | reverse primer | CCGACCATTATTCCTGCTGC |
| *MPC1* | forward primer | AGATGAGTAAGCGGCCATCTGCCT |
|  | reverse primer | AGCCGAGAGTTGGTTTGGGGAT |
| *MPC2* | forward primer | TGCTGCCAAAGAAATTGAGGCCG |
|  | reverse primer | GCACAGTGGATTGAGCTGTGCTGA |
| *β-actin* | forward primer | TCGCCTTTGCGATCCG |
|  | reverse primer | ATGATCTGGGTCATCTTCTCG |

**Table S2.** Primary antibodies used in the study.

| **Antibody** | **Company (Cat. No.)** | **Working dilutions** |
| --- | --- | --- |
| MPC1 | abcam (ab74871) | WB: 1/1000; IHC 1/200 |
| MPC2 | abcam (ab236584) | WB: 1/1000; IHC 1/200 |
| DRP1 | abcam (ab184247) | WB: 1/1000 |
| FIS1 | abcam (ab156865) | WB: 1/1000 |
| MFF | abcam (ab129075) | WB: 1/1000 |
| MFN1 | abcam (ab221661) | WB: 1/1000 |
| MFN2 | abcam (ab205236) | WB: 1/1000 |
| OPA1 | abcam (ab157457) | WB: 1/1000 |
| MARCH5 | Proteintech (12213-1-AP) | WB: 1/1000; IHC 1/200; IF 1/200 |
| MARCH5 | Cell signaling technology (#19168S) | IP: 1/100 |
| β-actin | Proteintech (20536-1-AP) | WB: 1/1000 |

**Table S3. Correlation between the expression of MPC1 and clinicopathologic features of 205 ovarian cancer patients.**

| Variables | No. of cases (%) | MPC1 expression | | *P* value |
| --- | --- | --- | --- | --- |
|  |  | Low | High |  |
| All | 205 (100%) | 102 | 103 |  |
| Age |  |  |  |  |
| <55 | 126 (61.5 %) | 66 | 60 | 0.390 |
| >=55 | 79 (38.8%) | 36 | 43 |  |
| Stage |  |  |  |  |
| I | 109 (53.2%) | 50 | 59 | 0.683 |
| II+III | 96 (46.8%) | 52 | 44 |  |
| Tumor size (cm) |  |  |  |  |
| <8 | 92 (44.9%) | 38 | 54 | **0.035** |
| >=8 | 113 (55.1% ) | 64 | 49 |  |
| Lymphatic invasion |  |  |  |  |
| No | 182 (88.8%) | 86 | 96 | **0.049** |
| Yes | 23 ( 11.2%) | 16 | 7 |  |
| Distant metastasis |  |  |  |  |
| No | 193 (94.1%) | 99 | 94 | 0.134 |
| Yes | 12 (5.9%) | 3 | 9 |  |


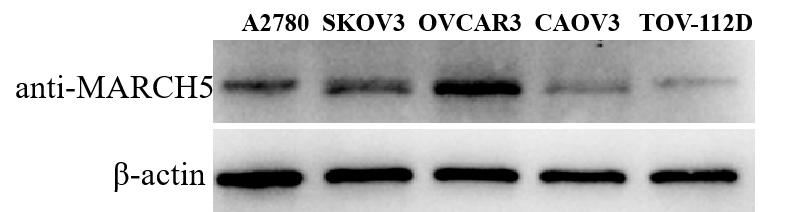


**Figure S1:** Western blot analysis showing the expression levels of MARCH5 in the five OC cell lines.
